# Harmful Effects of Smoking Cannabis: A Cerebrovascular and Neurological Perspective

**DOI:** 10.3389/fphar.2019.01481

**Published:** 2019-12-06

**Authors:** Sabrina Rahman Archie, Luca Cucullo

**Affiliations:** ^1^Department of Pharmaceutical Sciences, Texas Tech University Health Sciences Center, Amarillo, TX, United States; ^2^Center for Blood Brain Barrier Research, Texas Tech University Health Sciences Center, Amarillo, TX, United States

**Keywords:** oxidative stress, cannabis, recreational, drug, abuse, cerebrovascular, neurodegenerative, stroke

## Abstract

Apart from being used as a medicine, cannabis or marijuana is the most widely abused recreational drug all over the world. The legalization and decriminalization of cannabis in Canada and various states of USA may be the underlying reason of the widespread popularity of it among young population. Various studies have reported about the relationship between cannabis use and different detrimental effects like cardiovascular, cerebrovascular, and neurological complications among different age groups. Specifically, the young population is getting adversely affected by this, harmful yet, readily accessible recreational drug. Although the mechanism behind cannabis mediated neurological and cerebrovascular complications has not been elucidated yet, the results of these studies have confirmed the association of these diseases with cannabis. Given the lack of comprehensive study relating these harmful complications with cannabis use, the aim of this narrative literature review article is to evaluate and summarize current studies on cannabis consumption and cerebrovascular/neurological diseases along with the leading toxicological mechanisms.

## Introduction

Cannabis, commonly termed as marijuana, weed, pot, and ganja, is the most widely used illicit recreational drug around the world (Thomas et al., 2014; Wolff and Jouanjus, 2017). It is extracted from the natural plant *Cannabis sativa* and among more than 60 cannabinoids, tetrahydrocannabinol is one of the major active ingredients of cannabis (Atakan, 2012). In addition to the natural source, the use of synthetic cannabis (SC) named as spice, K2 or Kronic has gained popularity during the last decade (Wolff and Jouanjus, 2017). In spite of using cannabis in medicinal purposes as antioxidant, anticonvulsant, anti-inflammatory, and neuroprotective, the detrimental effects of it cannot be denied (Ford et al., 2017). Acute and chronic use of cannabis is associated with different harmful effects on central nervous system and peripheral system including hyperemesis syndrome, impaired coordination and performance, anxiety, suicidal/tendencies, psychotic symptoms and mood disorders, cannabis withdrawal symptoms, exacerbation of psychotic disorders, neurocognitive impairment, cardiovascular, neurological, respiratory, cerebrovascular, peripheral vascular diseases (Thomas et al., 2014; Karila et al., 2014), pneumomediastinum, pneumothorax, pneumopericardium, bullous lung disease, increased risk of chronic obstructive pulmonary disease, desquamated interstitial disease, and appearance of brown pigmented macrophages (Milroy and Parai, 2011).

Despite having serious effects of marijuana in human health, its use has been legalized in Canada and different states of USA. The Canadian Parliament passed Bill C-45, the *Cannabis Act* to legalize and regulate the production, distribution, and consumption of cannabis on June 19, 2018, and its legalization started effective from October 17, 2018 (Crepault, 2018). In case of US, marijuana use has been approved in 34 states for medical purposes (State Medical Marijuana Laws, 2019) and in 10 states for recreational purposes (Marijuana Overview, 2019).

Even though cannabis has medicinal benefit, recent studies have shown that chronic cannabis inhalation may be associated with cerebrovascular disease such as ischemic stroke (Thanvi and Treadwell, 2009) although the underlying mechanism between stroke and cannabis use has not been strongly established yet. Moreover, the hemorrhagic stroke occurrence has been rarely reported in different studies (Goyal et al., 2017). Several neurological disorders such as cognitive dysfunction, behavioral problems, memory, attention deficiency, structural, and functional changes in brain have been observed in different studies related to cannabis exposure (Chadwick et al., 2013; Battistella et al., 2014; Broyd et al., 2016; Szutorisz and Hurd, 2018). Increased use of cannabis or cannabinoids is associated with several complications related to different organs including the neurological and cerebrovascular system in human body. Due to this, exhaustive studies need to be performed to establish the possible link between cannabis inhalation and neurological and cerebrovascular effect. Keeping the popularity of cannabis use in mind, the aim of this review article is to list the neurological and cerebrovascular effects of marijuana inhalation including the probable mechanisms related to these effects.

### Methodology

Three biomedical literature databases, PubMed, Google Scholar, and ScienceDirect were searched up to July 2019. The search was conducted using “cannabis,” “cannabinoid,” “cannabidiol,” “delta-9-THC,” “endocannabinoids,” “CB1 receptor,” “CB2 receptor,” “cerebrovascular system,” “Blood Brain Barrier,” “stroke,” “neurological disease,” “neuroprotective effect,” “oxidative stress.” Articles dealing with medical use of cannabis were excluded as the aim of our review article is based on harmful effects of cannabis inhalation on cerebrovascular and neurological system. Case reports based on cannabis inhalation and cerebrovascular diseases were also searched and evaluated for inclusion in this review. Peer-reviewed articles presenting results of experimental studies in animal models and population-based studies were analyzed and presented in this review paper.

### What Are Cannabinoids?

**Figure 1:**
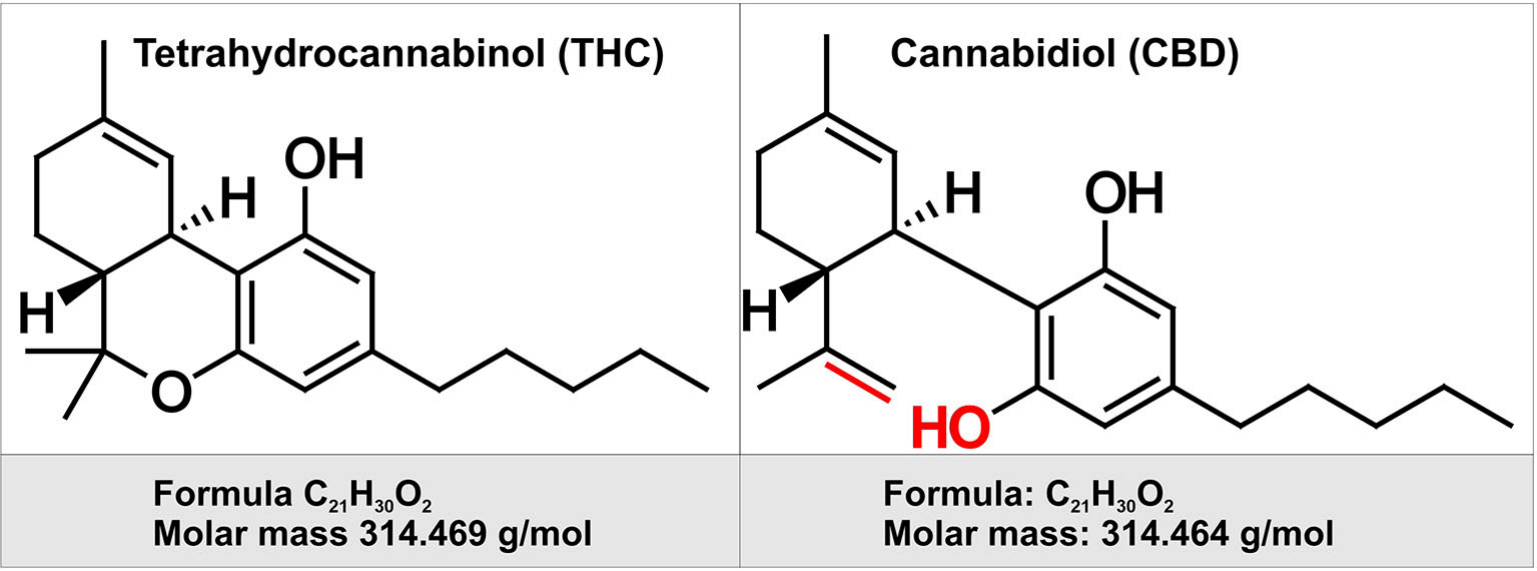
Chemical structure of two of the major cannabinoids contained in Marijuana. Depicted on the left is the chemical structure of tetrahydrocannabinol (THC). THC is the principal psychoactive constituent of cannabis. THC acts as a partial agonist at the cannabinoid receptor CB1 (primarily located in the brain and spinal cord as well as CB2 receptor expressed in cells of the immune system. On the right is depicted the chemical structure of cannabidiol (CBD). By contract with THC, CBD does not have any psychotropic effects, but appears to have some have anti-anxiety and anti-psychotic properties. CBD has a lower affinity for both CB1 and CB2 receptor when compared to THC. Highlighted in red are the chemical structure differences between CBD and THC.

Cannabinoids (CBs) are a group of chemical compounds which have varying affinity to cannabinoid receptors. Generally, cannabinoids can be classified into three groups namely, phytocannabinoids (isolated from natural source, *C. sativa*), synthetic cannabinoids, and endocannabinoids (Richter et al., 2018). Although cannabinoids can be extracted naturally from the plants, it can also be cultivated indoors using hydroponic and artificial lighting system nowadays. It was first cultivated in Central Asia however gradually it was brought to cultivate all over the world (Singh et al., 2018). Generally, it is collected from three strains of cannabis plant named *C. sativa*, *Cannabis indica*, and *Cannabis ruderalis* which differ in the content and amount of the active ingredients called Δ^9^-tetrahydrocannabinol (THC) and cannabidiol (CBD) (Booth et al., 2017) (see **Figure 1**) and among these strains, the highest proportion of THC is present in *C. sativa*. THC was isolated as one of the first phytocannabionoids (Gaoni and Mechoulam, 1964). In the plant *C. sativa*, this molecule is present as tetrahydrocannabinolic acid which is then decarboxylated to THC (Richter et al., 2018). Usually, the buds and the leaves of the cannabis plants contain the highest amount of psychoactive ingredient, THC. This THC can be taken up from dried buds and leaves by smoking as well as it can be taken in other forms for instance edibles, waxes, oils, liquid incense, or vapor for both medical and recreational uses. For medical purpose, cannabis has been used to treat nausea and vomiting due to chemotherapy, neuropathic pain related to cancer and advanced neurological disorders (Singh et al., 2018). However, the popularity of cannabis use lies in its recreational purposes. Therefore, in spite of being listed as a schedule 1 substance according to the Section 202 of the Controlled Substances Act of 1970 by Drug Enforcement Administration of USA, the use of cannabis has been legalized or decriminalized in different states of USA (Singh et al., 2018).

Besides natural sources, THC compounds can be synthesized for both medical and recreational uses. Since 1985, two synthetic THC compounds named dronabinol and nabilone have been using in USA in capsule forms for treating nausea, vomiting, and weight loss related to chemotherapy and acquired immunodeficiency syndrome. Recently, an oral solution of dronabinol has been approved by US FDA for treating anorexia and nausea/vomiting associated with acquired immunodeficiency syndrome and chemotherapy respectively. Besides, highly potent cannabinoids and cannabimimetics can be synthesized illegally by altering the structure of THC in numerous ways for recreational purposes which have already gained popularity among the users due to its potency, longer duration, and the failure of conventional drug screening tests to recognize the compounds (Gurney et al., 2014).

### Chemical Composition of *C. Sativa*

More than 421 chemicals are present in *C. sativa* of which 61 chemicals are cannabinoids. During cannabis smoking, over 2,000 compounds including hydrocarbon, nitrogenous compounds, amino acids, fatty acid, sugar etc. are produced by pyrolysis and all of these substances are responsible for different pharmacological as well as toxicological properties of cannabis (Sharma et al., 2012).

### Pharmacokinetics of Cannabinoids

Around 20%-70% of THC can be delivered through smoking (Adams and Martin, 1996). Smoking a 500 to 1,000 mg cannabis cigarette provides a THC dose of 0.2–4.4 mg where a pharmacologic effect of cannabis requires a dose of 2–22 mg. The THC level in the brain typically represents only ∼1% of the administered dose and usually corresponds to 2–44 μg.

THC is absorbed and reaches high blood concentration rapidly after inhalation through lungs (Vandevenne et al., 2000). Due to extensive lipid solubility and large volume of distribution, THC has a long biological half-life (18 h to 4 days) (Adams and Martin, 1996; Ashton, 2001) and gets distributed in adipose tissue, liver, lung, and spleen (Chiarotti and Costamagna, 2000; Sharma et al., 2012). Hydroxylation of THC generates psychoactive compound, 11-hydroxy ▵9_tetra hydrocannabinol (11-OH-THC), and further oxidation of this compound yields inactive compound, 11-nor-9-carboxy-▵9-tetrahydrocannbinol (THCCOOH) which is important for diagnostic purposes (Musshoff and Madea, 2006). The bioavailability of ▵9 THC depends on several factors including inhalation depth, duration of puff, and breath hold. It has been found that, the systemic bioavailability of THC is around 23–27% in heavy users whereas the value is 10–14% in case of occasional users (Sharma et al., 2012). The time to reach maximum plasma concentrations for ▵9 THC, 11-OH-THC, and THCCOOH is 8, 15, and 81 min after onset of smoking, respectively. On the other hand, systemic absorption of THC is relatively slow after oral ingestion compared to inhalation. In case of oral ingestion, the peak plasma concentration of ▵9 THC was observed after 1–2 h of ingestion which could be further delayed by few hours in some cases (Lemberger et al., 1971; Hollister et al., 1981). The oral bioavailability of ▵9 THC may be reduced by 4–12% by extensive hepatic metabolism (Owens et al., 1981).

Regular cannabis use can be defined as taking cannabinoids 10 to 19 times monthly, whereas heavy use can be termed as using 20 times in a month. However, both regular and heavy use of cannabis are related to several chronic health problems including anxiety, depression, and neurocognitive alterations (Hall and Degenhardt, 2009).

### Endocannabinoid System

**Figure 2:**
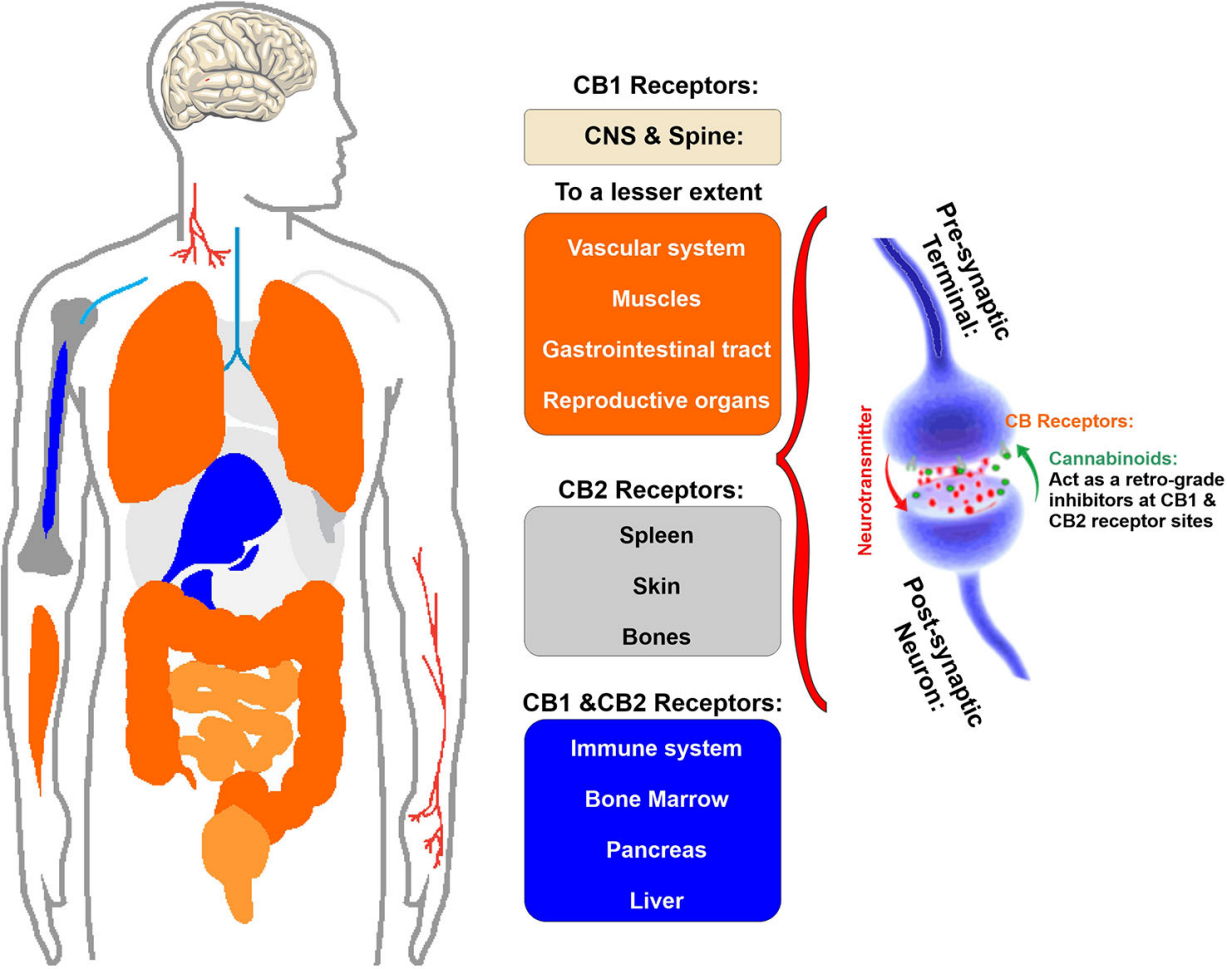
Schematic illustration of the primary location of CB1 and CB2 receptor. Note that CB1 receptor are primarily located in the brain and spinal cord and to a much lesser extent there are also present in the gastrointestinal tract, reproductive organs as well as muscles and vascular system. CB2 receptors are primary located in spleen, skin, and bones as well as the immune cells.

**Figure 3:**
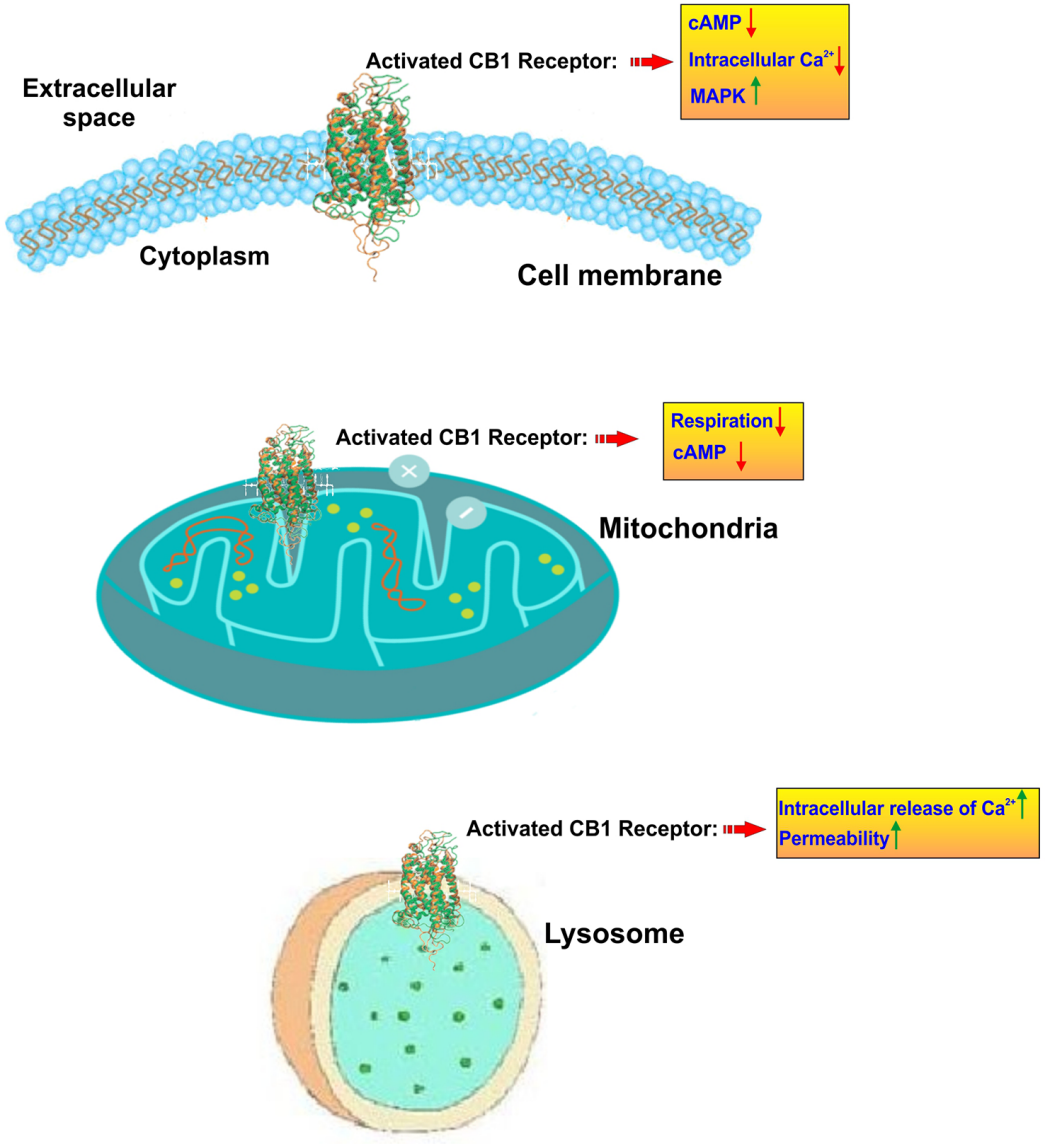
Subcellular localization and activity of CB1 receptors. CB1 receptors are primary located on the cell membrane where their activation lead to inhibition of adenylate cyclase and a resulting reduction of cyclic AMP. In parallel CB1 activation promotes the upregulation of mitogen-activated protein kinase (MAPK) which is involved in directing cellular responses to mitogens, heat shock, osmotic stress, and proinflammatory stimuli (e.g. cytokines). At the mitochondrial level, CB1 activation leads to inhibition of mitochondrial respiration and production of cAMP. CB1 receptors are also present at the level of lysosomes where they prompt a release of calcium from these internal storage units and increase the intracellular calcium levels. Lysosome permeability is also increased.

Cannabinoids interact directly with our body through a complex system named endocannabinoid system which helps to maintain homeostasis of body by regulating metabolism, intercellular communication, appetite, and memory, immune, and pain responses. This endocannabinoid system (ECS) consists of two types of receptors namely CB1 and CB2 (Zou and Kumar, 2018) (see **Figure 2**). CB receptors mainly belong to the G-protein coupled receptor (GPCR) family, having inhibitory function on the cyclic adenosine monophosphate (cAMP) pathway through intracellular signal transduction (Richter et al., 2018). Although CB1 receptors are scattered all over the body, these are present predominantly in anatomical regions of the brain (Grotenhermen, 2005) related to memory, anxiety, cognition, pain sensory, motor coordination, endocrine function (Herkenham et al., 1990; Adams and Martin, 1996). CB1 receptors have the inhibitory action on cAMP production which is facilitated by the activation of adenyl cyclase inhibitor subunit of G proteins (G_i/0_ proteins). Ultimately, this leads to an inhibition of N and P/Q type calcium currents and an activation of A type, inwardly rectifying potassium currents and mitogen activated protein kinase (Sierra et al., 2015) (see **Figure 3**).

On the other hand, CB2 receptors are located in peripheral nervous system and immune system and the primary function of this receptor is anti-inflammatory activity through initiating an immune response to reduce inflammation as well as tissue damage (Turcotte et al., 2016). Also, it plays a pivotal role in the immune suppressive action of the cannabinoids (Sharma et al., 2012) (see also **Figure 2**).

The psychoactive agent of cannabis, THC binds with the cannabinoid 1 (CB1) receptor in the brain and the non-psychoactive component, CBD is the most likely to interact with cannabinoid 2 (CB2) receptor and exert their activities. Therefore, cannabis is used in medical purposes to reduce inflammation, relieve pain, and decrease seizures (Rivera-Olmos and Parra-Bernal, 2016; Perucca, 2017).

Moreover, phytocannabinoid Δ^9^ THC can also bind with other binding sites including the transient receptor potential cation-channel subfamily V member 1 (TRPV1) and peroxisome proliferator activator receptors (PPARs) (O’Sullivan, 2016). Also, GPR 18 receptor has been proposed as a potential cannabinoid receptor (Console-Bram et al., 2014).

### Physiology of Cerebrovascular System

The word cerebrovascular consists of two parts: “cerebro” means large part of the brain and “vascular” refers to arteries and veins. Thus, the cerebrovascular system refers to the blood vessels through which blood is carried out to and from the brain. The cerebrovascular anatomy has an endocranial and exocranial component that can be further divided into the anterior and posterior circulation based on the contribution of blood flow through the internal carotid arteries (ICAs) and vertebral arteries, respectively (Hendricks et al., 2018).

Blood-brain barrier (BBB), a part of the neurovascular unit (NVU), is a unique, dynamic, physical, and biochemical regulatory boundary/barrier which restricts and regulates the exchange of molecules, ions, and cells between the peripheral circulation and central nervous system (CNS) (Yang et al., 2019) and maintains cerebral homeostasis precisely as well (Abdullahi et al., 2018). It transports essential nutrients which are required for the normal metabolism of the brain cells (Sivandzade and Cucullo, 2018). The BBB is primarily composed of endothelial cells, pericytes, and astrocytes restricting the communication between blood and the brain parenchyma (Abbott et al., 2010). As a result, the BBB plays a pivotal role in restraining the entry of neurotoxic plasma components, xenobiotics, blood cells, and pathogens in the brain (Winkler et al., 2011) thus protecting the CNS from inflammation, injury, and different types of diseases (Daneman and Prat, 2015). On contrary, the BBB also limits drug delivery into the brain to treat different neurological diseases (Abbott, 2013; Kaisar et al., 2017).

Disruption of BBB is associated with different neurological disorders for instance stroke, MS, epilepsy, Alzheimer’s disease, traumatic brain injury etc. (Abbott et al., 2010; Abbott and Friedman, 2012). Breakdown of BBB has been observed in many functional imaging of human patients and postmortem brain samples in various neurological diseases. This breakdown not only causes edema and disrupts ionic homeostasis but also results in altered signaling and immune infiltration. As a consequence, BBB breakdown leads to neuronal dysregulation and ultimately to neuronal degeneration (Daneman and Prat, 2015).

### Effect of Cannabis Inhalation on the Cerebrovascular System

Studies on acute neurovascular events related to cannabis use have appeared as early as 1964 (Mohan and Sood, 1964). Stroke is the fifth leading cause of death in USA (Vijayan et al., 2019) and recent preclinical studies, population based study, case reports, and reviews have portrayed the correlation of cannabis (both naturally and synthetically derived) to ischemic and hemorrhagic cerebrovascular diseases (Rose et al., 2015) which clearly suggests that cannabis plays a pivotal role in the etiology of cerebral stroke (Wolff et al., 2015a).

### Animal Studies

To examine the effect of cannabinoids on blood circulation as well as reactive vasodilation or vasoconstriction, particularly focusing on the cerebral vascular bed, several studies on rat, mouse, rabbit, cat, and pig models were performed (Richter et al., 2018). It was found from various studies that, both vasodilation and vasoconstriction have been observed following administration of Δ^9^ THC, 11-OH-THC, AEA, and AM-404 in rodents. However, vasodilation has been observed after administering Abn-CBD and O-1966. On the other hand, all perfused cerebral vessels reacted with wall relaxation in case of large mammals (Richter et al., 2018).

From studies in isolated cerebral vessel of rabbit and cat, it has been found that cannabinoid mediated vasodilation through CB1 receptor by inhibiting Ca^+^ influx in cerebral arterial muscle cell as well as possibly through arachidonic acid metabolism (Ellis et al., 1995; Gebremedhin et al., 1999). Additionally, three studies on rat models (Bloom et al., 1997; Stein et al., 1998; Iring et al., 2013) partially indicated the occurrence of vasoconstriction which may result in hypoperfusion by a reduction of CBF and could be a mechanism of neuronal death through ischemia (Richter et al., 2018). Other studies involving rat and pig models have demonstrated the cerebrovascular dilation due to cannabinoids perfusion (Hillard et al., 2007; MacIntyre et al., 2014; Su et al., 2015).

No study reported any possible mechanism underlying vasoconstrictive effect due to cannabis. It has also been suggested that, vasodilating effect could be beneficial or detrimental depending on the time of vasodilation after CNS insult. As vasodilation reduces the peripheral resistance and increases CBF, this may serve as a protective mechanism and increase oxygen supply in case of a cerebral insult. This protective mechanism may be beneficial in early stages of ischemia. However, if it happens in a later stage, it might augment the recuperation of cerebral function (Richter et al., 2018). As these studies were conducted in different variety of animal models involving different experimental setup, cannabinoid molecules, and their respective doses, it is difficult to draw any conclusion (Richter et al., 2018).

### Case Reports

A total of 107 case reports involving cannabis (both raw and synthetic) intake and neurovascular complications have been studied since 1964 to 2019 (**Table 1**) and it has been postulated from all these reports that there may be a link between these two events although this correlation has not been established strongly yet.

**Table 1:** List of case reports related to neurovascular complications after natural and synthetic cannabinoid use (according to year; 1964–2019).

No of patient	Sex/Age	Types of cannabinoids	Risk factors	Neurovascular complication	Year	Reference
01	M/18	Cannabis	C	Undetermined	1964	(Wolff et al., 2013)
01	M/19	Cannabis	C	Undetermined	1977	(Garrett et al., 1977)
02	M/28/27	Cannabis	C	Undetermined	1987	(Cooles and Michaud, 1987)
02	M/34/32	Cannabis	C(2)/T(2)	IS	1991	(Zachariah, 1991)
01	M/30	Cannabis	C/T/A	IS	1992	(Barnes et al., 1992)
01	M/22	Cannabis	C/T/A	TIA and IS	1996	(Lawson and Rees, 1996)
01	M/29	Cannabis	C/T/A	TIA and IS	1997	(McCarron and Thomas, 1997)
03	M/18/26/30	Cannabis	C(3)/T(2)	TIA	2000	(Mouzak et al., 2000)
01	M/23	Cannabis	C/T/A	IS	2001	(Mesec et al., 2001)
01	M/18	Cannabis	C/T	IS	2001	(Marinella, 2001)
01	M/33	Cannabis	C/T	IS	2002	(Alvaro et al., 2002)
01	M/27	Cannabis	C/T	IS	2002	(Russmann et al., 2002)
01	M/37	Cannabis	C/T/L	IS	2004	(Finsterer et al., 2004)
03	M/15/16/17	Cannabis	C(3)	IS	2004	(Geller et al., 2004)
01	M/50	Cannabis	C/T	TIA and IS	2005	(Haubrich et al., 2005)
01	M/36	Cannabis	C/A	IS	2005	(Mateo et al., 2005)
02	M/26/29	Cannabis	C	IS	2006	(Mateo et al., 2006)
01	F/41	Cannabis	C	IS	2006	(Noskin et al., 2006)
01	M/27	Cannabis	C/T/A	IS	2007	(Termote et al., 2007)
01	M/46	Cannabis	C/A/MoA/H/L	IS	2007	(Calabrese et al., 2007)
01	F/34	Cannabis	C/T	ICH	2008	(Renard and Gaillard, 2008)
01	M/46	Cannabis	C/T	IS	2008	(Koopman et al., 2008)
01	M/22	Cannabis	C	IS	2009	(Bal et al., 2009)
01	F/45	Cannabis	C/T/A	IS	2010	(Duchene et al., 2010)
01	M/26	Cannabis	C/T	IS	2011	(Leblanc et al., 2011)
01	M/24	Cannabis	C/T	IS	2011	(Trojak et al., 2011)
01	M/40	Cannabis	C/T/A	IS	2011	(Maguire et al., 2011)
01	M/18	Synthetic Cannabinoid: Kronic purple haze	C	SAH	2011	(Kamat et al., 2012)
01	M/33	Cannabis	C/T	IS	2012	(Renard et al., 2012)
01	M/32	Cannabis	C/T	IS	2012	(Barbieux et al., 2012)
02	M/27 F/62	Cannabis	C(2)/T(1)/MoA(1)/A(1)	SAH	2012	(Marder et al., 2012)
14	M/22/37/44/49/50/56/58/59/61/63 F/27/28/44/52	Cannabis	C(14)/T(13)/H(3)/A(3)	IS	2012	(Singhal et al., 2011)
01	M/41	Synthetic cannabinoid: Spice; daily use	C	SAH	2012	(Ray et al., 2013)
01	M/16	Cannabis	C	IS	2013	(El Scheich et al., 2013)
02	F/23/53	Cannabis	C(2)	IS	2013	(Robert et al., 2013)
02	F/22	Synthetic cannabinoid: K2	C	IS	2013	(Bernson-Leung et al., 2014)
	F/26	Synthetic cannabinoid: peak extreme	C/T			
02	M/26	Synthetic: K2 (JWH-018)	C	IS	2013	(Freeman et al., 2013)
	F/19	Synthetic: K2 (JWH-018)	C/T			
01	M/42	Cannabis	C	TIA and IS	2014	(Tsivgoulis et al., 2014)
01	M/27	Cannabis	C/A	IS	2014	(Santos et al., 2014)
01	M/26	Cannabis	C	IS	2014	(Oyinloye et al., 2014)
01	F/32	Cannabis	C/MoA	IS	2014	(Nouh et al., 2014)
01	M/34	Cannabis	C/T/A	IS	2014	(Ellis et al., 1995)
01	M/22	Cannabis	C	IS	2014	(Baharnoori et al., 2014)
01	M/23	Cannabis	C/T	IS	2014	(Inal et al., 2014)
01	M/33	Synthetic cannabinoid: WTF (XLR-11 analyzed in the product)	C	IS	2014	(Takematsu et al., 2014)
01	M/21	Cannabis	C	IS	2015	(Whitlock et al., 2015)
01	F/50	Cannabis	C/H/L/MoA	IS	2015	(Yau et al., 2015)
03	M/28/33 F/35	Cannabis	C(3)/T(2)/A(2)	IS	2015	(Ntlholang et al., 2015)
01	M/19	Cannabis	C	IS	2015	(Domi et al., 2015)
01	M/38	Cannabis	C/T/A	ICH and IS	2015	(Ince et al., 2015)
14	M/19/20/21/29/31/36/37/38/44 F/21/24/26/31/33	Cannabis	C(14)/T(14)/MoA(2)/D(4)/H(3)/A(5)	IS	2015	(Wolff et al., 2015)
01	M/45	Synthetic cannabinoid:	C/T/H/D	SAH	2015	(Sherpa et al., 2015)
02	M/31	Synthetic cannabinoid: Spice (K2), XLR-11 analyzed in the packet	C	SAH/ICH	2015	(Rose et al., 2015)
	F/25	Synthetic cannabinoid: Spice (K2) + marijuana		SAH/IS		
01	M/33	Cannabis	C/T	IS	2016	(Jamil et al., 2016)
01	M/25	Cannabis	C/A	IS	2016	(Tirkey and Gupta, 2016)
01	M/48	Synthetic cannabinoid: Chronic Bonzai	C/T	IS	2016	(Degirmenci et al., 2016)
01	M/15	Synthetic: Bonzai (heavy smoker for 2.5 years)	C/T	IS	2016	(Keskin et al., 2016)
01	F/32	Synthetic cannabinoid: chronic use	C/T	IS	2016	(Raheemullah and Laurence, 2016)
01	M/25	Munakka or Bhang tablets	C/A	IS	2016	(Tirkey and Gupta, 2016)
01	M/25	Cannabis joints	C/T/A	HS	2017	(El Mesbahy et al., 2017)
01	F/14	Cannabis	C/A	IS	2017	(Volpon et al., 2017)
01	F-51	Cannabis	C	ICH	2017	(Shere and Goyal, 2017)
01	M/27	Raw cannabis	C	ICH	2017	(Atchaneeyasakul et al., 2017)
01	M/37	Cannabis	C/T	IS	2018	(Anghelescu, 2018)
01	M/36	Synthetic cannabinoid, K2	C/T/A	IS	2018	(Faroqui et al., 2018)
01	F/47	Synthetic cannabinoid	C	IS	2018	(Jung et al., 2018)
01	M/37	Marijuana	C/T	IS	2019	(Sharma et al., 2019)

M, male; F, female; C, cannabis; SC, synthetic cannabinoids; T, tobacco; A, alcohol; L, dyslipidemia; MoA, migraine without aura; H, hypertension; FD, diabetes; IS, ischemic stroke; SAH, subarachnoid haemorrhage; ICH, intracranial haemorrhage; TIA, transient ischemic attack.

From these studies we can conclude that among the 107 neurovascular cases, almost 84% were ischemic stroke related to cannabis or cannabinoid use (both natural and synthetic). Considering **Table 1** it is found that, young population is experiencing alarming number of neurovascular complications due to recreational use of cannabis. Statistically, around 14% and 36.4% of reported cases were involved in teenager (below 20 years) and young people (21–30 years) respectively. Besides, about 26% of the reported patients in these case reports were aged between 31 to 40 years. However, the occurrence of neurovascular complication among middle aged and older people was significantly lower compared to the young and adult population with a value of 5.6% (aged between 51–60 years) and 2.80% (aged between 61–70 years). This data clearly indicates that, young people are severely affected by neurovascular diseases as they consume cannabis in higher amount compared to older people. Although some studies have been able to establish the possible correlation between cannabis use and ischemic stroke occurrence, only 11% of total reports have demonstrated the incident of hemorrhagic stroke due to cannabis exposure (Fonseca and Ferro, 2013; Wolff and Jouanjus, 2017). Moreover, compilation of these case reports indicates that along with cannabis or cannabinoids use, other risk factors like alcohol, tobacco, dyslipidemia, migraine without aura, hypertension etc. also act as prodromal factors for the onset of cerebrovascular diseases.

Interestingly, it can also be noted from these case reports that the occurrence of neurovascular diseases such as stroke has drastically increased after 2010. Widespread availability of cannabis or synthetic cannabinoids and its legalization across the world may be the underlying reasons behind this.

### Population-Based Study

Due to the alarming effect of cannabinoids on public health, several population-based studies have been performed to correlate the relation between cannabinoid exposure as well as cerebrovascular diseases. Various studies demonstrated that cannabinoids may act as a risk or prognostic factor for cerebrovascular diseases such as stroke (Westover et al., 2007; Barber et al., 2013; Hemachandra et al., 2016; Rumalla et al., 2016a; Rumalla et al., 2016b). **Table 2** summarizes all the findings from population-based analysis, conducted between 2000 and 2015.

**Table 2:** Summary of population-based analysis related to cannabis use, conducted between 2000 and 2015.

Study type	Duration	Study design	Outcome	Strength	Limitation	Ref
Cross-sectional	2000–2003	N = 3,148,165 patients’ age: 18–44	Cannabis was considered as a risk factor for IS (OR 1.76, 95% CI 1.15–2.71) and for hemorrhagic stroke (OR 1.36, 95% CI 0.9–2.06)	-Large sample size, other risk factors e.g. amphetamine, cocaine were considered	-Unable to distinguish between primary and secondary or recurrent strokes.	(Westover et al., 2007)
				-Study was conducted in a database representative of hospitalized conditions in all but the smallest rural hospitals in Texas.	- Possibility of misclassification of variables in a database of International classification of diseases, ninth revision, clinical modification (ICD-9-CM)–coded discharge diagnoses.	
Cross-sectional	2004–2007	N = 200, Mean age at admission was 28.0 years (95% CI 26.7, 29.3).	Four cerebrovascular accidents in patients below 40 were found related to cannabis use	-Accurate detection of cannabis related hospitalization.	-Study was conducted in a restricted geographical area	(Jouanjus et al., 2011)
				-Other AEs e.g. psychiatric disorders, acute intoxication, respiratory and cardiovascular disorders were recorded.	-Small sample size.	
					- Difficulty in assessing precise epidemiological reference data in a context of illicit drug consumption.	
Case-control	2009	Patients’ age: 18–55	15.6% of patients had IS/TIA, cannabis use was related to increased risk of IS/TIA (OR: 2.30, 95% CI 1.08–5.08)	-Evidence of an association between a lifestyle that includes cannabis and tobacco, and IS	-Association between cannabis and IS/TIA independent of tobacco could not be confirmed.	(Barber et al., 2013)
				- Use of a control cohort	- Other factors e.g. socioeconomic or employment status, alcohol and/or drug use, could not be measured.	
				-Urinary drug screens were conducted.	-Absence of extensive comparisons between patients and controls	
Case series	2006–2010	N = 1,979, mean age: 34.3	1.8% cardiovascular complications reported, three of which were cerebrovascular complications (acute cerebral angiopathy, transient cortical blindness and cerebral artery spasm.	-Long study period	-lack of data due to underreporting of cases.	(Jouanjus et al., 2014)
				-Cardiovascular disorders were focused.	Some cases were not exhaustively informed.	
					- Toxicologic analyses were available in only 37% of cases. Lack of data on cardiac or vascular disease history, body mass index.	
Cross-sectional	1999–2002	20–24 years (N = 2,383), 40–44 years (N = 2,525), and 60–64 years (N = 2,547)	153 stroke/TIA cases (2.1%). Cannabis users (n = 1,043) had 3.3 times the rate of stroke/TIA (95% CI 1.8–6.3, *p* < 0.001). Elevated stroke/TIA was specific to participants who used cannabis weekly or more often (IRR 4.7, 95% CI 2.1–10.7)	-Large sample size based on different age range.	-Lack of detailed history of participants’ cannabis use.	(Hemachandra et al., 2016)
				-Able to adjust for a wide variety of lifestyle and health factors that were related to stroke/TIA.	-Only correlate past year cannabis use with lifetime occurrence of stroke.	
					-Unable to control tobacco consumption concurrently with cannabis, family history of stroke/TIA, hyperlipidemia, or other drug use (e.g. recreational stimulant use)	
Cohort	2004–2011	N = 2,496,166 Patients’ age: 15–54	Greater incidence of IS among cannabis users compared to non-users (RR 1.13, 95% CI 1.11–1.15)	-Long study period	-Only primary diagnoses of AIS were included which may resulted in under-diagnosis of AIS patients.	(Rumalla et al., 2016a)
				-Association of other risk factors e.g. tobacco, cocaine, amphetamine was also considered.	-Unable to comprise any dose-dependent mechanisms of cannabis use due to the limitations of the ICD-9-CM coding system.	
					- lack of data on preadmission functional status and severity in the NIS, constrains adjustment for AIS severity.	
Cohort	2004–2011	Cannabis users: N = 2,496,165 Non-cannabis users: N = 116,163,453 Patients’ Age: 15–54	Aneurysmal SAH incidence was slightly increased in the cannabis cohort compared to non-cannabis cohort (RR 1.07, 95% CI 1.02–1.11)	-Long study period	- Possibility of inaccuracy of diagnoses and procedural codes used to identify SAH patients from the NIS database.	(Rumalla et al., 2016b)
				- Use of the NIS database.	- Possibility of misclassification and under-classification of drug use using secondary ICD-9-CM codes.	
				-Large sample size	- No data related to the time from last drug use to aSAH was available.	
					- Assessment of preadmission functional status and severity of aSAH at admission was not possible to determine using NIS.	
Cohort	2009–2014	N = 725 ICH	Cannabinoid use in 8.6% ICH. No link was found between cannabinoid use and specific characteristics of ICH. CB+ patients had milder ICH presentation and less disability at discharge.	-Use of international, multicenter, observational, collaborative database.	-Lack of data on time of cannabis consumption.	(Di Napoli et al., 2016)
Cohort	2010–2015	N = 108 Patients’ age ≥18 years	25.9% with CB+ and delayed cerebral ischemia was diagnosed in 50% of CB+. CB+ was independently associated with delayed cerebral ischemia (OR, 2.68; 95% CI, 1.03–6.99; *P* = 0.01) and poor outcome (35.7% versus 13.8%; *P* = 0.01) in SAH patients.	-Long study period	-Lack of patient care uniformity.	(Behrouz et al., 2016)
				- UDS was performed.	-Limited ability to identify and include other relevant clinical features, including cardiopulmonary comorbidities, infections, recurrent aneurysmal rupture, hydrocephalus, and seizures.	
					-Unable to differentiate new cannabis use from residual drug excretion, considering that cannabinoids may persist in the urine for several days/weeks.	
					-Unable to differentiate chronic use from single-episode cannabis consumption.	
					-Unable to eliminate the possibility of false positive or negative UDS completely.	

OR, odds ratio; CI, confidence interval; RR, relative risk; ICH, intracranial hemorrhage; SAH, subarachnoid hemorrhage; TIA, transient ischemic stroke; IS, ischemic stroke; CB+, cannabinoid user; UDS, urine drug screen.

The result from these large sample size studies provide information on the temporal relationship between cannabis use and cerebrovascular complications like intracerebral hemorrhage (ICH), subarachnoid hemorrhage (SAH), and ischemic strokes (IS). Along with cannabinoids, other predominant risk factors were also considered in the assessments however, these studies have several limitations. This include lack of consideration for the high lipid solubility of cannabis metabolites which helps them to persist in fatty tissues, therefore they may be detected in the urine weeks after the initial use (Mateo et al., 2005) and this may lead to erroneous result interpretation.

### Probable Mechanism Associated With Cannabis-Mediated Neurovascular Diseases

It is evident from various studies that, consumption of cannabinoids through inhalation and combustion, is associated with the occurrence of cerebral infarcts (Garrett et al., 1977; Wolff and Jouanjus, 2017). Natural cannabis and synthetic cannabinoids may act as possible trigger for reversible intracranial vasoconstriction (Wolff et al., 2015a) which along with severely reduced cerebral blood flow (CBF) could be a major prodromal factor to neuronal death by ischemia (Wolff et al., 2014; Wolff and Jouanjus, 2017).

**Figure 4:**
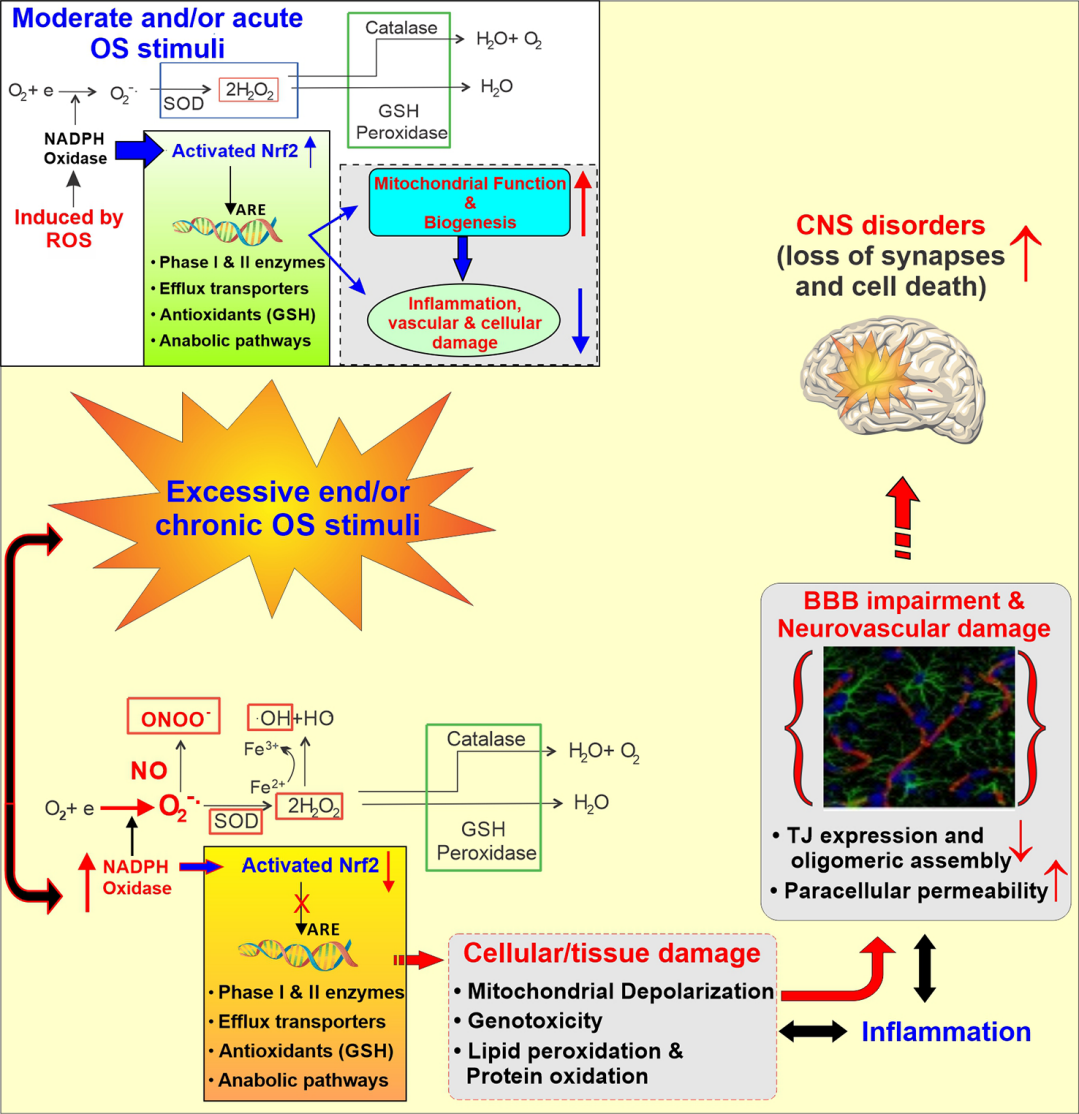
Schematic illustration of the Activation of the cellular antioxidative response system under normal and stress condition. Under normal conditions, the response to injury is adaptive, designed to restore homoeostasis and to protect the cell from further injury. In response to excessive oxidative stress stimuli, NADPH oxidase is activated, producing an excess of O_2_^‑^ which in the presence of nitric oxide (.NO; also abundant in CS and release in response to IR) results in formation of peroxinitrite (ONOO^‑^). Furthermore, the excess of H_2_O_2_ leads to the formation of hydroxyl radicals (OH; Fenton’s reaction). The unchecked OS leads then to mitochondrial depolarization, lipid peroxidation, DNA fragmentation and inflammation which at the cerebrovascular level can cause BBB damage and ultimately facilitate the onset of CNS diseases.

Different types of mechanisms might be involved in the development of stroke in cannabis users including orthostatic hypotension with the secondary impairment of the CBF autoregulation, altered cerebral vasomotor function, supine hypertension, and fluctuations in blood pressure, cardioembolism with atrial fibrillation, vasculopathy and vasospasm (Singh et al., 2012; Wolff et al., 2013; Hirapara and Aggarwal, 2016), cerebral artery luminal stenosis, increased carboxyhemoglobin level, RCVS, and angiopathy (Goyal et al., 2017). Although none of these mechanisms have been fully vetted to explain the association between use of cannabis and stroke occurrence, reversible cerebral vasoconstriction triggered by cannabis could be the most convincing theory to explain it (Wolff and Jouanjus, 2017). It was shown in different case reports that, cannabis use was associated with reversible multifocal intracranial arterial stenosis (Noskin et al., 2006; Calabrese et al., 2007; Koopman et al., 2008; Renard and Gaillard, 2008; Wolff et al., 2011; Marder et al., 2012; Tsivgoulis et al., 2014; Nouh et al., 2014; Yau et al., 2015; Wolff et al., 2015a). Along with this, another eye-catching mechanism to explain the relationship between cerebrovascular complications and cannabis use could be the cellular effect of cannabis on brain mitochondria. A recent *in vivo* study conducted on mice has shown that THC inhibited the complexes I, II, and III of the respiration chain of mitochondria and increased the amount of hydrogen peroxide production (Wolff et al., 2015b). This strongly suggests that ROS production and therefore, oxidative stress, could be the linking mechanism between cannabis use and stroke. This well cope with current knowledge that oxidative stress and inflammation are established prodromal factors for the onset of stroke and other neurological disorders in humans (Chen et al., 2011) (see also **Figure 4**).

### Preclinical Studies Related to the Effects of Cannabinoids and Cannabinoid Receptor on Stroke Outcome

Different preclinical studies demonstrated the effects of cannabinoids and cannabinoid receptors on stroke outcome, One of particular interest is the fact that cannabinoids not only decreased infarct volume following an ischemic stroke (both transient and permanent occlusion models), but also improved early and late functional outcome (England et al., 2015). Furthermore activation of the endogenous cannabinoid signaling pathway as indirectly demonstrated by a study conducted on CB1 receptor knockout mice which exhibited increased mortality, severe infarct size, and neurological deficits after transient local cerebral ischemia, reduced cerebral blood flow, and increased N-methyl-d-aspartate (NMDA) neurotoxicity when compared to wild type (Parmentier-Batteur et al., 2002).

Post-stroke inflammatory responses can be reduced by CB2 ligands whereas, activation of CB1 receptors promotes chemical hypothermia. Both processes result in a reduced stroke infarct volume (Leker et al., 2003; Murikinati et al., 2010). Specifically, activation of CB1 receptor activation reduces glutamate release (Hayakawa et al., 2007), allied excitotoxicity (Shen et al., 1996) and increased cerebral blood flow (Parmentier-Batteur et al., 2002). On the other hand, CB2 receptors activation results in reduced pro-inflammatory cytokines release, neutrophil recruitment (Murikinati et al., 2010; Zarruk et al., 2012) and adhesion of leukocyte to cerebral vessels (Zhang et al., 2007).

Another study demonstrated that CB2 receptor plays a major role in driving neuroblast migrations as well as subsequent neurogenesis in the peri-infarct cortex after experimental stroke in mice which positively impact stroke outcome. It was also suggested that, endocannabinoid tone is essential for this process by promoting migration of neuroblasts toward the injured brain tissue which leads to increased number of new cortical neurons. As a result, motor functional recovery is increased which is beneficial for improving the outcome of aged patients as well as reducing their disabilities after chronic stroke (Bravo-Ferrer et al., 2017).

Even though these preclinical studies suggest a neuroprotective action of cannabinoids and cannabinoid receptors on stoke outcome, the matter is far from being set. In fact, conflicting results remain (Rivers-Auty et al., 2014; Garberg et al., 2016). Rivers-Auty et al. demonstrated that, CB2R agonist GW405833, cannot improve brain damage related to hypoxia-ischemia in rat models (Rivers-Auty et al., 2014). Another study conducted by Garberg HT et al. did not find any significant neuroprotective action of cannabinoids after hypoxia-ischemia in piglets (Garberg et al., 2016). Finally, most of the neuroprotective effect of cannabinoids related preclinical studies did not evaluate stroke outcomes with behavioral studies however, behavioral studies are integral part of stroke outcome research (Alamri et al., 2018). Investigators tried to correlate stroke outcomes with histological scores. Since histological improvement does not confirm long term post stroke benefits (Clarkson et al., 2010), further studies with proper behavioral outcomes is required to clarify these contradictory findings (Rivers-Auty et al., 2014). Furthermore, there are no human studies to confirm or refute these in data and properly assess the post-stroke effects of cannabinoids as neuroprotectant.

### Neurological Effect of Cannabis or Cannabinoids

Cannabis based medications for instance nabiximols and THC have therapeutic potential against some symptoms associated with neurological diseases such as multiple sclerosis, chronic pain (Cohen et al., 2019). Moreover, CBD, CBDV, and ▵9 THCV have been shown to have activity against epilepsy (Scutt and Williamson, 2007; Dennis et al., 2008; Jones et al., 2010; De Petrocellis et al., 2011; Ma et al., 2008).

Exogenous cannabinoids such as CBD and nabilone are also found to have therapeutic activity in psychiatric disorders including schizophrenia, posttraumatic stress disorder (PTSD) and general and social anxiety (Cohen et al., 2019). Although cannabinoid-based drugs have shown some therapeutic activities against neurological and psychiatric disorders the effect of cannabis on the neurological system cannot be denied. It has been demonstrated from various *in vivo* studies that THC is responsible for inducing dose-dependent toxicity as well as causing structural changes in those parts of brain which are rich in CB1 receptors. These receptors are located primarily in cerebellum, hippocampus, amygdala, prefrontal cortex, and striatum (Lawston et al., 2000; Downer et al., 2001; Burns et al., 2007). However, studies conducted on human brains assessing the long term cannabis use and related brain structural changes do not fully confirm these findings although changes in the density of gray or white matter have been reported in different regions of frontal and parietal lobes (Matochik et al., 2005; Churchwell et al., 2010; Gruber et al., 2011). This disconnect between *in vivo* and human studies might be due to the different sample characteristics, inter-individual variabilities related to past history of drug use, consumption rate, psychological problems, and differences in the experimental methodology (Batalla et al., 2013). Moreover, some studies demonstrated changes in the hippocampus/parahippocampal complex and amygdala (Matochik et al., 2005; Yucel et al., 2008; Zalesky et al., 2012; Goyal et al., 2017) and a recent one reported a significant reduction in gray matter in the CB1 receptors enriched regions of brain including media l temporal cortex, temporal pole, parahippocampal gyrus, insula, and orbitofrontal cortex when regular cannabis users were compared to groups of occasional users. These regions of the brain control motivation, emotion, and affective processing (Battistella et al., 2014). Various studies reported that, adolescents can experience persistent deficiency in different cognitive functions including attention, memory, and processing speed due to chronic cannabis use (Chadwick et al., 2013; Broyd et al., 2016; Szutorisz and Hurd, 2018). It has been found from neuropsychological tests and advanced imaging techniques that, learning process of adolescents can be affected by cannabinoid use as well (Rivera-Olmos and Parra-Bernal, 2016).

Additionally, fatal brain development can also be affected by cannabis exposure during pregnancy which may ultimately result in impaired vision and coordination, larger intermittent attention, as well as behavioral problems in children at later phase (Wu et al., 2011).

Also, different psychiatric diseases (**Table 3**) including schizophrenia, bipolar disorder, social anxiety, and suicidal thought are found to be higher in cannabis users compared to non-users (Mental Health, 2017).

**Table 3:** Adverse psychiatric consequence of cannabis use (NIDA, 2019a).

Acute (present during intoxication)	Persistent (lasting longer than intoxication, but may be temporary)	Long-term (total effects of repeated use)
Impairment of short-term memory	Impaired learning and coordination	Potential for marijuana addiction
Impairment of cognitive functions	Sleep disorder	Impairment of learning and memory with potential loss of IQ
Impaired coordination and balance		Increased risk of other drug and alcohol use disorders
Anxiety, paranoia		Increased risk of schizophrenia in people with genetic vulnerability
Psychosis (uncommon)		

These detrimental effects of cannabinoid may vary from person to person because of genetic variability. Besides, these detrimental effects can also depend on the age of the user. For instance, the exposure of adolescents to cannabinoids leads to severe memory impairment compared to adult (Jouroukhin et al., 2019).

Several studies have demonstrated the potential role of gene variation on the development of psychosis due to cannabis use (NIDA, 2019a). It has been found that risk of psychosis among the daily cannabis users carrying a specific variant of AK21 gene is seven times higher compared with those who use it infrequently or never used (Di Forti et al., 2012). Another study revealed an increased risk of psychosis among adults who carry a specific variant of the gene for *catechol-O-methyltransferase* (COMT) enzyme and used cannabis during adolescence. This enzyme can degrade different neurotransmitters for instance, dopamine and norepinephrine (Caspi et al., 2005).

Interestingly, cannabis use has also been shown to worsen the condition of schizophrenic patients. Cannabis can cause an acute psychotic reaction at high doses, in non-schizophrenic people who are cannabis users, although this fades as the drug effects wears off (NIDA, 2019a).

### Probable Mechanisms Associated With Cannabis Mediated Neurological Diseases

Although the mechanism behind THC induced cognitive and behavioral dysfunction is yet to be established, recent studies conducted on mice model have demonstrated that these detrimental effects are facilitated by astrocyte CNR1 (Han et al., 2012; Chen et al., 2013). Moreover, THC may activate nuclear factor κB signal as well as upregulate cyclooxygenase-2 (COX-2) which may result in elevated release of glutamate by astrocytes (Bezzi et al., 1998).

Interestingly, all the cannabis users do not experience cognitive impairment which clearly suggests the impact of genetic vulnerability on detrimental effects of cannabis (Blanco et al., 2016; Levine et al., 2017; Hasin, 2018). Likewise, several preclinical studies in genetically mutated mice model for psychiatric disorders showed higher effect of THC on memory (O’Tuathaigh et al., 2010; Long et al., 2013; Tantra et al., 2014; Ballinger et al., 2015; Segal-Gavish et al., 2017) although the underlying mechanism of the effect of genetic mutation on cognitive function has not been delineated yet (Jouroukhin et al., 2019). A recent study reported that genetic predisposition and THC exposure synergistically activates the NF-κB-COX-2 signal in astrocytes. This activation results in excessive glutamate secretion and decreased immunoreactivity of parvalbumin-positive presynaptic boutons around pyramidal neurons of the CA3 area of the hippocampus as well as impaired memory. It has been suggested from this research that, COX-2 inhibitors can prevent these cognitive deficits which may act as a potential target for future studies (Jouroukhin et al., 2019).

### Cannabis and Oxidative Stress

It is well established that oxidative stress (OS) is associated with vascular endothelial dysfunction in a causative and dose dependent manner. Current scientific opinion considers the exposure to reactive oxygen species (ROS; e.g. H_2_O_2_, epoxides, nitrogen dioxide, peroxynitrite- ONOO^‑^, etc.), and OS-mediated pathways leading to inflammation (Ahmed et al., 2019) and cellular/tissue damage to play a major role in the pathogenesis of cerebrovascular and neurological disorders like stroke, and Alzheimer’s and BBB impairment (Sajja et al., 2018). At the cerebrovascular level OS promotes oxidative damage and BBB breakdown *via* tight junction (TJ) modification as well as activation of proinflammatory pathways (Pun et al., 2009). Under normal conditions, ROS are converted into less reactive molecules by superoxide dismutase (SOD), catalase, and glutathione peroxidase (GSH-Px) as well as scavenged by endogenous antioxidants including vitamins (such as ascorbic acid and α-tocopherol) (Chiu et al., 2008; Hossain et al., 2011). Activation of the Nuclear factor erythroid 2-related factor (NRF2), a redox-sensitive transcription factor which, in turn, promotes the activation of several biological systems encompassing anti-inflammatory molecules, antioxidants, drug metabolizing enzymes (including cytochrome P450s), and free radical scavengers, also plays a critical protecting role against OS(136-138). However, chronic exposure to OS stimuli [such as that to tobacco smoke (Prasad et al., 2017)] can overwhelm these protective mechanisms and/or compromise their functionality (Prasad et al., 2017; Kaisar et al., 2018).

From the point of view of oxidative stress, several studies suggest that smoking marijuana is not much different than smoking tobacco. Sarafian et al. have previously shown that marijuana cigarettes promote the formation of ROS while lowering the intracellular levels of glutathione (Sarafian et al., 1999). In addition, other investigators found that THC, the main psychoactive component in the cannabis, acts as a potent promoter of OS and inflammation, thus appearing as a risk factor for the onset of ischemic stroke (Wolff et al., 2015b). On the other hand, there are also evidences that non-psychoactive CBDs, can have neuroprotective effects by reducing the reactivity of microglial cells, and transmigration of leukocytes (through downregulation of chemokines, interleukin-1, and vascular cell adhesion molecule-1) (Mecha et al., 2013). *In vitro* studies using amyloid-beta-stimulate PC12 neurons CBD could inhibit the activity of inducible nitric oxide synthase, thus preventing the production of nitric oxide and reducing OS (Esposito et al., 2006). By contrast, *in vivo* studies to assess the protective effect of cannabis treatment against OS development and nigrostriatal cell injury induced by intrastriatal injection of rotenone did not produce any significant result (Omar, 2015). Unfortunately, there are contrasting results concerning the oxidative and antioxidative property of cannabinoids. Some of these controversial results could be attributed to the length of exposure such as acute vs. long term chronic exposure. As for the overall pro-oxidative effect of smoking marijuana, it is very likely that ROS are generated as a byproduct of combustion rather than a direct effect of cannabinoids. Similarly to tobacco smoke where most of the oxidative stress is generated by the combustion of tobacco rather than exposure to nicotine which can also promotes OS but to a much lower extent (Naik et al., 2014).

## Discussion

Cannabis or marijuana is the most widely used recreational drug and around 181 million people use it around the world ^2^. Even though cannabis is used for treating inflammation, nausea seizure, pain, mental disorder, addiction (NIDA, 2019b) movement problem, Alzheimer’s (Mack, 2000), uncontrolled use of cannabinoids may have severe detrimental effects. Now-a-days cannabis use among young people especially teenagers has been increased drastically as a recreational element. National Institute of Drug Abuse reported that, 13.90% of 8^th^ graders, 32.60% of 10^th^ graders, and 43.60% of 12^th^ graders are prevalent to marijuana or hashish, according to the data of 2018 (NIDA, 2018). It has also been found that, cannabis use among college students in US remains at the highest level in last three decades. Around 38% of full time college students and 41% of non-full time college students, who are aged 19–22 used cannabis at least once in one year (Schulenberg et al., 2018; Marijuana use among US college students remains at highest level in three decades, 2018). This data clearly indicates the widespread use of cannabis among youth and it is the high time to elucidate the consequence of cannabis use in human. Although the direct effect of cannabis exposure and health consequences is still unknown, numerous case reports, population-based studies as well as animal studies demonstrated the potential link between cannabis use and neurovascular as well as neurological diseases. It is evident from various studies that, recreational use of cannabinoids is related to both cardio and cerebrovascular events such as ischemic and hemorrhagic stroke (Goyal et al., 2017) and neurological diseases for instance structural and functional changes in brain, cognitive dysfunction as well as behavioral disorders (Chadwick et al., 2013; Battistella et al., 2014; Broyd et al., 2016; Szutorisz and Hurd, 2018). Unfortunately, both cerebrovascular and neurological disorders are found to be higher in young population as they are the main consumer.

Although the underlying mechanism behind cannabis use and occurrence of cerebrovascular diseases has not been elucidated yet, the handful of case reports and preclinical studies on animal model highlighted provide some plausible insights. These includes (but not limited to) reversible cerebral vasoconstriction (Wolff et al., 2015a), ROS generation inducing oxidative stress (although, similar to tobacco smoke ROS could be generated as a byproduct of marijuana combustion rather than a specific effect of cannabinoids) (Chen et al., 2011), cerebral artery luminal stenosis, cerebral auto-dysregulation, cardioembolism, reversible cerebral vasoconstriction syndrome (RCVS), angiopathy (Goyal et al., 2017). A very recent report showed that, genetic modifications as well as age of consumers play a pivotal role in developing neurological disorders (Jouroukhin et al., 2019). However, additional and more specific studies will be necessary to determine their relevant contribution to the onset of cerebrovascular and neurological disorders.

To the best of our knowledge, a very few studies have been performed to understand the mechanism of detrimental effect of cannabis on both neurology and the BBB. Since the BBB restricts the communication between blood and brain parenchyma and maintains cerebral homeostasis, damage of BBB results in neuronal dysregulation and degeneration. Therefore, it is evident the studying the effects of cannabis and particularly chronic exposure to it, should be considered a major target for future studies.

## Author Contributions

SA conceived the study and prepared the drafting of the manuscript. LC assisted with the drafting of the manuscript and preparation of the figures. LC also oversaw the entire project and provided funding support. All authors reviewed the manuscript.

## Funding

This work was supported by the National Institutes of Health/National Institute on Drug Abuse 2R01-DA029121-01A1 and National Institutes of Health/Food and Drug Administration 1R01-OD026234 to LC.

## Conflict of Interest

The authors declare that the research was conducted in the absence of any commercial or financial relationships that could be construed as a potential conflict of interest.

## Abbreviations

cAMP, Cyclic adenosine monophosphate; BBB, Blood-Brain Barrier; CBs, Cannabinoids; CBD, Cannabidiol; CBF, Cerebral blood flow; CNS, Central nervous system; COMT, Catechol-O-methyltransferase; COX-2, Cyclooxygenase-2; ECS, Endocannabinoid system; GPCR, G-protein coupled receptor; GSH-Px, Glutathione peroxidase; ICH, Intracerebral hemorrhage; IS, Ischemic strokes; OS, Oxidative stress; NMDA, N-methyl-d-aspartate; NRF2, Nuclear factor erythroid 2-related factor; NVU, Nuclear factor erythroid 2-related factor; PTSD, Nuclear factor erythroid 2-related factor; RCVS, Reversible cerebral vasoconstriction syndrome; ROS, Reactive Oxygen Species; SAH, Subarachnoid hemorrhage; SC, Synthetic cannabis; SOD, Superoxide dismutase; THC, Δ^9^-tetrahydrocannabinol; TJ, Tight junctions; 11-OH-THC, 11-hydroxy Δ9_Tetra hydrocannabinol; THCCOOH, 11-nor-9-carboxy-Δ9-tetrahydrocannbinol; TRPV1, Transient receptor potential cation-channel subfamily V member 1; PPARs, Peroxisome proliferator activator receptors.
